# Applied precision cancer medicine in metastatic biliary tract cancer

**DOI:** 10.1007/s12072-020-10020-6

**Published:** 2020-02-25

**Authors:** H. Taghizadeh, L. Müllauer, R. Mader, G. W. Prager

**Affiliations:** 1 Department of Medicine I, Clinical Division of Oncology, Medical University of Vienna, Spitalgasse 23, 1090 Vienna, Austria; 2 Comprehensive Cancer Center Vienna, Vienna, Austria; 3 Clinical Institute of Pathology, Medical University Vienna, Vienna, Austria

**Keywords:** Precision cancer medicine, Biliary tract cancer, Cholangiocarcinoma, Molecular aberrations, Molecular profiling, Molecular oncology, Gene sequencing, Immunohistochemistry, Targeted therapy, Tumor board

## Abstract

**Introduction:**

Advanced therapy-refractory biliary tract cancer (BTC) has poor prognosis and constitutes a major challenge for adequate treatment strategies. By mapping the molecular profiles of advanced BTC patients, precision cancer medicine may provide targeted therapies for these patients.

**Objective:**

In this analysis, we aimed to show the potential of PCM in metastatic BTC.

**Methods:**

In this single-center, real-world retrospective analysis of our PCM platform, we describe the molecular profiling of 30 patients diagnosed with different types of metastatic BTC. Tumor samples of the patients were examined using a 161-gene next-generation sequencing panel, immunohistochemistry (IHC), and fluorescence in situ hybridization for chromosomal translocations.

**Results:**

In total, we identified 35 molecular aberrations in 30 patients. The predominant mutations were *KRAS* (*n* = 8), *TP53* (*n* = 7), *IDH2* (*n* = 4), and *IDH1* (*n* = 3) that accounted for the majority of all molecular alterations (62.86%). *BRAF* mutations were observed in two patients. Less frequent alterations were noted in *ARID1A*, *CTNNB1*, *ESR1*, *FBXW7*, *FGFR2*, *MET*, *NOTCH2*, *PIK3CA*, *PTCH1*, *SMAD4*, and *SRC1*, each in one case. *FGFR* fusion gene was detected in one patient. No mutations were detected in eight patients. IHC revealed EGFR and p-mTOR expression in 28 patients. Applying these results to our patients, targeted therapy was recommended for 60% of the patients (*n* = 18). One patient achieved stable disease.

**Conclusions:**

PCM is a feasible treatment approach and may provide molecular-guided therapy recommendations for metastatic BTC.

**Electronic supplementary material:**

The online version of this article (10.1007/s12072-020-10020-6) contains supplementary material, which is available to authorized users.

## Introduction

Biliary tract cancer (BTC) is a highly malignant and fatal cancer that arises from the biliary epithelium in the bile duct, gall bladder, or ampulla of Vater. The term BTC encompasses several entities: gallbladder carcinoma, distal cholangiocarcinoma, perihilar cholangiocarcinoma, intrahepatic cholangiocarcinoma, and ampullary carcinoma. BTC is a relatively rare cancer with an incidence of about 2/100,000 in the Western world [1]. Independent of the subtype, the current established standard first-line treatment for metastasized BTC is the combination of gemcitabine with cisplatin, as reported by the ABC-02 trial [2].

For a long time, no second-line treatment was established. Only recently, in May 2019, Lamarca et al. [3] introduced a second-line treatment for metastasized BTC, which is a combination of oxaliplatin, folinic acid, and fluorouracil and was tested in the ABC-06 phase III trial. However, after the failure of these two therapies, therapeutic options for advanced BTC are limited, and their use is not supported by prospective, randomized clinical trials.

BTC is a slow-growing malignancy that causes non-specific symptoms. Thus, this disease entity is often diagnosed only in the advanced stages. Due to late symptomatology, the paucity of effective treatments, molecular diversity, and poor understanding of the complex molecular mechanisms and pathways, BTC has a dismal prognosis [4]. In stage IV, BTC has a poor median survival prognosis of 11.7 months despite therapeutic efforts [2].

In recent years, efforts have been made to progressively individualize therapy options in specific cancers. In a few particular cancers, treatment with tyrosine kinase inhibitors or immunotherapeutic agents tailored to the individual is possible, for example trastuzumab in HER2-positive breast cancer or gastric cancer, imatinib in Ph + CML or in KIT + GIST, and pazopanib and sunitinib in advanced renal cell carcinoma [5].

Emerging techniques, such as profiling tumor molecular alterations and mutations, identifying molecular targets amenable to specific treatments, and developing drug treatments specific to an individual patient, have created the potential for novel and effective therapies. This approach is known by a number of different names, including individualized, stratified, tailored, or precision cancer medicine (PCM). The main rationale of PCM is to match a therapeutic agent to its corresponding molecular target to allow a precise treatment tailored to a specific patient. It aims to achieve a better and more sustained response than do more generic treatments, without damaging healthy cells and tissues.

In this study, we sought to map the molecular profiles of advanced, pretreated, and mainly relapsed BTC and to specifically target the detected molecular alterations.

## Materials and methods

### Patients and design of the precision medicine platform

We conducted a retrospective subgroup analysis of 30 patients with metastasized BTC, who were enrolled and profiled in our special PCM platform of molecular oncological diagnostics and therapy (MONDTI) of the Comprehensive Cancer Centre of the Medical University of Vienna (CCC-MUV), Vienna, Austria. Patients with pretreated, metastasized BTC who had progressed to all standard treatment options were eligible for inclusion in MONDTI, provided that their archival tissue samples were available. Patients needed to have an Eastern Cooperative Oncology Group (ECOG) performance status of 0 or 1. MONDTI is not a clinical trial, but it intends to provide the possibility of a targeted therapy to patients for whom no standard antitumoral treatment is available yet. All patients had to provide informed consent before being enrolled in MONDTI. Furthermore, the Institutional Ethics Committee of the Medical University of Vienna approved this subanalysis (No. 1039/2017).

### Tissue samples

Formalin-fixed, paraffin-embedded tissue tumor samples were sent to or retrieved from the archive of the Department of Pathology, Medical University Vienna.

### Cancer gene panel sequencing

DNA was extracted from paraffin-embedded tissue blocks with a QIAamp Tissue KitTM (Qiagen, Hilden, Germany), and 10 ng DNA per sample was utilized for sequencing. The DNA library was generated by multiplex polymerase chain reaction with the 161-gene next-generation sequencing panel of Oncomine Comprehensive Assay v3 (Thermo Fisher Scientific, Waltham, MA, USA).

### Immunohistochemistry (IHC)

IHC was performed using 2-μm-thin tissue sections read by a Ventana Benchmark Ultra stainer (Ventana, Tucson, Arizona, USA). The following antibodies were applied: anaplastic lymphoma kinase (ALK) (clone 1A4; Zytomed, Berlin, Germany), CD20 (clone L26; Dako), CD30 (clone BerH2; Agilent Technologies, Vienna, Austria), epidermal growth factor receptor (EGFR) (clone 3C6; Ventana), estrogen receptor (clone SP1; Ventana), human epidermal growth factor receptor 2 (HER2) (clone 4B5; Ventana), HER3 (clone SP71; Abcam), *C*-*kit* receptor (KIT) (clone 9.7; Ventana), MET (clone SP44; Ventana), phosphorylated mammalian target of rapamycin (p-mTOR) (clone 49F9; Cell Signaling Technology, Danvers, Massachusetts, USA), platelet-derived growth factor alpha (PDGFRA) (rabbit polyclonal; Thermo Fisher Scientific), PDGFRB (clone 28E1, Cell Signaling Technology), programmed death-ligand 1 (PD-L1) (clone E1L3N; Cell Signaling Technology), progesterone receptor (clone 1E2; Ventana), phosphatase and tensin homolog (PTEN) (clone Y184; Abcam), and ROS1 (clone D4D6; Cell Signaling Technology).

To assess the immunostaining intensity for the antigens EGFR, p-mTOR, PDGFRA, PDGFRB, and PTEN, a combinative semiquantitative score for immunohistochemistry was used. The immunostaining intensity was graded from 0 to 3 (0 = negative, 1 = weak, 2 = moderate, 3 = strong). To calculate the score, the intensity grade was multiplied by the percentage of corresponding positive cells: (maximum 300) = (% negative × 0) + (% weak × 1) + (% moderate × 2) + (% strong × 3).

The immunohistochemical staining intensity for HER2 was scored from 0 to 3 + (0 = negative, 1 + = negative, 2 + = positive, 3 + = positive) pursuant to the scoring guidelines of the Dako HercepTestR from the company Agilent Technologies (Agilent Technologies, Vienna, Austria).

For PD-L1, the tumor proportion score was calculated which is the percentage of viable malignant cells showing membrane staining.

ALK, CD30, CD20, and ROS1 stainings were classified positive or negative based on the percentage of reactive tumor cells, however without graduation of the staining intensity. In ALK- or ROS1-positive cases, the presence of a possible gene translocation was evaluated by fluorescence in situ hybridization

### Multidisciplinary boards (molecular tumor boards for PCM)

After analysis by an experienced molecular pathologist, the molecular profile of each tumor sample was discussed within the multidisciplinary tumor boards (MTB) that were held every other week.

Members of the board included molecular pathologists, radiologists, clinical oncologists, biostatisticians, and basic scientists. Targeted therapy was chosen on the basis of the individual tumor profile and comprised tyrosine kinase inhibitors, checkpoint inhibitors (e.g., anti-PD-L1 monoclonal antibodies), and growth factor receptor antibodies with or without endocrine therapy. Treatment decisions by the multidisciplinary team were prioritized according to the level of evidence from high to low according to phase III to phase I trials.

If more than one targetable alteration was detected, a combination therapy encompassing as many molecular targets as possible was chosen, considering the toxicity profile of each drug and the potential mutual interactions of the drugs. Given that patients received all standard treatment options for their specific malignancy before being enrolled in the molecular profiling program, almost all matched targeted agents were recommended as off-label use. If the molecular profile met the inclusion criteria of a clinical trial for molecular targets that was ongoing in our cancer center, patients were preferentially asked if they wanted to participate in this trial.

### Descriptive statistics

For data description, we used measures of central tendency including the mean and median. We also used the method of frequency distribution to delineate the characteristics of the BTC patients

## Results

Thirty patients diagnosed with advanced, pretreated, and metastasized BTC were included from June 2013 to July 2019 in this specific analysis from the cohort of the PCM project MONDTI that has so far profiled 550 patients with various advanced and therapy-refractory cancer types. All the patients included in this analysis were Caucasians, including 16 male and 14 female. Twenty-four patients were diagnosed with intrahepatic cholangiocarcinoma (CCC), and six patients were diagnosed with distal CCC. The histological subtype of all patients was an adenocarcinoma. The median age at first diagnosis was 58.1 years, ranging from 34.9 to 74.9 years, and the median age at the time of molecular profiling was 59.7 years, ranging from 35.1 to 76.0 (Table 1).

**Table 1:** Patient characteristics (*n* = 30)

Patient characteristics	Number
Mean age at fist diagnosis (in years)	58.1
Mean age at molecular profiling (in years)	59.7
Male	16
Female	14
Caucasian	30
Intrahepatic cholangiocarcinoma	24
Extrahepatic cholangiocarcinoma	6
Received therapy prior to inclusion in the precision cancer medicine group	2–5
Therapy recommendations	18

In 16 patients, the tumor sample was obtained during surgical intervention. The median time period between initial diagnosis and surgery in resectable tumors was 7 weeks. The median turnaround time between surgery and molecular profiling of the tumor sample was 13 months (range 2–48 months). Fourteen patients had an unresectable cancer and underwent biopsy for diagnostic confirmation of BTC. Nine of them underwent a rebiopsy for the purpose of molecular profiling.

The median turnaround time between initiation of molecular profiling and discussion in MTB and therapy initiation for all 30 patients was 29 and 35 days, respectively.

Our platform for precision medicine was initiated in June 2013. From 2013 to 2017, the median turnaround time from molecular profiling to therapy initiation was at 44 days. Nine of them underwent a rebiopsy between 2018 and 2019 for the purpose of molecular profiling. For those nine patients, the median turnaround time from biopsy to discussion in MTB and to therapy initiation was 18 and 21 days, respectively.

At the time of molecular profiling, all the patients had advanced and therapy-refractory BTC in stage IV. Eleven patients had undergone surgical intervention. All patients had received systemic chemotherapy with the standard first-line treatment gemcitabine and a platinum-based agent. After the failure of platinum-based chemotherapy, nab-paclitaxel, either combined with capecitabine or gemcitabine, was administered as the second-line treatment to ten patients and third-line treatment to four patients. Fluoropyrimidine-based therapy was applied, either in combination with irinotecan or oxaliplatin or both agents, in four patients.

All the patients had distant metastasis, including eight patients with osseous metastases, four patients with peritoneal carcinomatosis, two patients with pleural carcinomatosis, and one patient with cerebral metastasis. In total, we identified 35 molecular aberrations in 30 patients: The predominant mutations were *KRAS* (*n* = 8), *TP53* (*n* = 7), *IDH2* (*n* = 4), and *IDH1* (*n* = 3) that accounted for the majority of all molecular alterations (62.86%). *BRAF* mutations were observed in two patients. One patient had the specific mutation *BRAF V600E* and was included subsequently in the ROAR trial. Less frequent alterations were seen in *ARID1A*, *CTNNB1*, *ESR1*, *FBXW7*, *FGFR2*, *MET*, *NOTCH2*, *PIK3CA*, *PTCH1*, *SMAD4*, and *SRC1*, each in one case. No mutations were detected in eight patients (Table 2). None of the patients had MSI-high BTC.

**Table 2:** Genomic profile of the BTC patients

Mutations	Number of mutations	Percentage
*KRAS*	8	23
*TP53*	7	20
*IDH2*	4	11
*IDH1*	3	9
*BRAF*	2	6
*ARID1A*	1	3
*CTNNB1*	1	3
*ESR1*	1	3
*FBXW7*	1	3
*FGFR2*	1	3
*MET*	1	3
*NOTCH2*	1	3
*PIK3CA*	1	3
*PTCH1*	1	3
*SMAD4*	1	3
*SRC*	1	3
	35	100

IHC revealed EGFR and p-mTOR expression in 28 patients. The EGFR median score was 210, and 16 patients had a high EGFR score of 200–300. The expression of p-mTOR was low, with a median score of 130, and six patients had a high p-mTOR score of 200–300. MET expression was reported in 18 patients, and PDGFRA and PTEN expression was less common and detected in 13 and five patients, respectively. Progesterone receptor and CXCR4 were expressed in three and two persons, respectively. PD-L1 expression was observed in six patients. One patient exhibited a fusion gene *FGFR2 (Exon 17)*–*OFD1* (Exon 3). HER2 was moderately expressed in three patients and strongly in one patient. FISH did not reveal any translocations. Targeted therapy was recommended for 60% of the patients (18/30) by the MTB, based on their individual molecular profile. The most frequently recommended agent was cetuximab, which was suggested for seven patients with *KRAS* wild type: as monotherapy in five cases and combined with everolimus in one and crizotinib in another case. Pembrolizumab and ponatinib were each considered in three and two patients, respectively. In one patient, everolimus was recommended in combination with an aromatase inhibitor for a patient with loss of PTEN, KRAS mutation, and PIK3CA mutation and expression of progesterone receptor. As mentioned above, in another patient everolimus was offered in combination with cetuximab in a patient with KRAS wild type and deficient PTEN.

Enasidenib, sorafenib, trastuzumab emtansine, trastuzumab, trametinib, and dabrafenib were each suggested in one patient. Table 3 depicts the rationale of the therapy suggestions. Eventually, seven patients received the recommended targeted therapy. One patient with a detected *BRAF V600E* mutation was included in the phase II ROAR trial, to be treated with dabrafenib and trametinib. One patient with EGFR expression was treated with cetuximab and achieved stable disease for 3 months. Two other patients were administered everolimus + cetuximab and everolimus + exemestane, respectively; however, they experienced progressive disease. Three patients received sorafenib, trastuzumab emtansine, and trastuzumab. However, they died before restaging could be performed. Sorafenib was applied as compassionate use. The expenses for everolimus, cetuximab, exemestane, trastuzumab, and trastuzumab emtansine were covered by the health insurance. Eleven patients died before a targeted therapy could be administered. Eight of them were included in the time interval from 2013 to 2017 when the median turnaround time was at 44 days from profiling to therapy initiation. Due to the long turnaround time in this time period, the patients rapidly deteriorated regarding their general health condition and unfortunately died before a targeted therapy application.

**Table 3:** Rationale for therapy recommendations

Therapeutic agent (trade name)	Targets	Overview of current FDA approval in different entities	Overview of current EMA approval in different entities	Number of recommended cases
Cetuximab (Erbitux^®^)	EGFR expression	CRC, HNSCC	CRC, HNSCC	Recommended in seven cases: In four cases as monotherapy In one case combined with crizotinib In one case combined with everolimus
Pembrolizumab (Keytruda^®^)	PD-1, hypermutability	Melanoma, NSCLC, HNSCC, HL, urothelial carcinoma, microsatellite instability-high cancer, gastric cancer, cervical cancer	Melanoma, NSCLC, HNSCC, HL, urothelial carcinoma	Recommended in three cases
Everolimus (Afinitor^®^)	mTOR expression	Breast cancer, PNET, RCC,	Breast cancer, RCC, neuroendocrine tumors of pancreatic, gastrointestinal, or lung origin	Recommended in two cases: In one case combined with cetuximab In one case combined with aromatase inhibitor
Sunitinib (Sutent^®^)	PDGFR, KIT, VEGFR, RET, GCSFR, FLT3	RCC, PDAC, GIST	RCC, PDAC, GIST	Recommended in one case
Ponatinib (Iclusig^®^)	BCR-ABL, FGFR1/2/3/4, VEGFR2, PDGFR	CML, Ph + ALL	CML, Ph + ALL	Recommended in two cases
Crizotinib (Xalkori^®^)	ALK, ROS1, HGFR, MET	ROS1 + or ALK + NSCLC	ROS1+ or ALK + NSCLC	Recommended in one case combined with cetuximab
Enasidenib (Idhifa^®^)	IDH2	AML	No approval	Recommended in one case
Dabrafenib/trametinib (Tafinlar^®^/Mekinist^®^)	BRAF V600E	BRAF V600E melanoma or NSCLC	BRAF V600E melanoma or NSCLC	Recommended in one case
Trastuzumab emtansine (Kadcyla^®^)	HER2	HER2 + breast cancer	HER2 + breast cancer	Recommended in one case
Trastuzumab (Herceptin^®^)	HER2	HER2 + breast cancer and gastric cancer	HER2 + breast cancer and gastric cancer	Recommended in one case
Sorafenib (Nexavar^®^)	PDGFR, RAF kinase, VEGFR,	HCC, RCC, thyroid carcinoma	HCC, RCC, thyroid carcinoma	Recommended in one case

*ABL* Abelson murine leukemia viral oncogene homolog 1, *AML* acute myeloid leukemia, *ALL* acute lymphatic leukemia, *BCR* breakpoint cluster region, *CML* chronic myeloid leukemia, *CRC* colorectal cancer, *EGFR* epidermal growth factor receptor, *EMA* European Medicines Agency, *FDA* Food and Drug Administration, *FLT3* fms-like tyrosine kinase 3, *GIST* gastrointestinal stromal tumor, *HCC* hepatocellular carcinoma, *HER2* human epidermal growth factor receptor 2, *HL* Hodgkin lymphoma, *HNSCC* head and neck squamous cell carcinoma, *NSCLC* non-small-cell lung carcinoma, *PD-1* programmed cell death protein 1, *PDAC* pancreatic ductal adenocarcinoma, *PDGFR* platelet-derived growth factor receptor, *Ph+* Philadelphia chromosome positive, *p-mTOR* phosphorylated mammalian target of rapamycin, *RCC* renal cell carcinoma, *RET* rearranged during transfection, *VEGFR* vascular endothelial growth factor

## Discussion

In this retrospective single-center analysis, we present the molecular profiling of 30 patients with BTC from the MONDTI cohort. Tumor tissue was obtained from all patients and characterized for molecular profiling. Subsequently, the molecular alterations of these patients were discussed in an MTB for PCM to evaluate the possibility of a molecular-based treatment independent of the tumor’s histological classification (tissue-agnostic treatment). Treatment recommendations were derived for 18 patients from the MTB. The drugs were carefully selected for individualized treatment, taking into account the patient’s clinical and treatment history, performance status, comorbidities, and concomitant therapies. The primary goal of our platform MONDTI is to provide targeted treatment suggestions in patients for whom no standard treatment is available. It does not intend to assess the therapeutic efficacy of the recommended agents.

Although this analysis shows that PCM is implementable in daily clinical routine, only one patient experienced a clinical benefit through this approach. One reason may be the turnaround time: A shorter turnaround time may help to commence treatment earlier with the targeted therapy and to control the cancer. Liquid biopsy may be a viable option to reduce the turnaround time, to monitor the disease, and to assess the response to therapy. Another reason may be the complexity of BTC. Our analysis shows that BTC exhibits genetic heterogeneity. This finding is consistent with the well-described extreme and complex intratumoral heterogeneity in BTC that occurs within the same tumor tissue; vascularization, proliferation, and subclones are all known to be highly variable. The pattern of genetic and epigenetic aberrations changes both spatially and temporally. Tumor biology at metastatic sites is different from that at the primary site and differs again at point of relapse. In addition, it is known that the therapy itself can influence and inform the clonal tumor evolution, by creating new driver mutations in subclones that become insensitive to drugs [6]. Since there was a median time period of 13 months between surgery and molecular profiling, the molecular landscape may had changed in the meantime.

The IHC methods used for our platform for PCM may contain pitfalls and limitations that could change or impact the IHC scorings and thus influence the therapy recommendations. One important pitfall is the time of ischemia of the retrieved tumor tissue sample during biopsy or surgical intervention. The cold ischemia time was shown to affect for instance estrogen and progesterone receptors. Further relevant parameters are thickness and size of tissue section that can affect signal altitude. Apart from these pre-analytical issues, the correct interpretation and assessment of the tissue sections are critical. There are no standardized and highly reliable methods for quantification of tissue protein content by IHC methods. Qualitative, semiquantitative, and quantitative scoring systems are employed to estimate the protein level. Manual and automatic systems are also applied. One critical factor for reproducibility is consistency in tissue scoring, particularly in manual assessment. Different pathologists may come to different conclusions. This heterogeneity can impede the correct interpretation and may influence the therapy recommendations in PCM. Awareness of these pitfalls and clear, specific, and distinct parameters and international standardization efforts can help to overcome these challenges [7].

The detected mutations and IHC scores observed in BTC in this analysis are in line with those observed in previous studies. Lowery et al. [8] performed a comprehensive genomic analysis of intrahepatic CCC and identified frequent mutations in *IDH1*, *ARID1A*, *BAP1*, and *TP53*–*FGFR* gene fusions. Reportedly, the genomic landscape of BTC varies with the location of the carcinoma. Intrahepatic CCC more frequently exhibits genomic aberrations in *IDH1*, *IDH2*, and *BAP1*, while extrahepatic CCC commonly harbors mutations in *KRAS*, *TP53*, and *SMAD4*. *IDH* mutations play a significant role in oncogenesis by producing the metabolite 2-hydroxyglutarate IDH1/2, which normally catalyzes the conversion of isocitrate to α-ketoglutarate. However, mutations in IDH1/2 result in a gain-of-function activity that leads to conversion of α-ketoglutarate to 2-hydroxyglutarate that does not have any physiological functions and accumulates in abundance within the cell. As an oncometabolite, it disrupts the function of the α-ketoglutarate-dependent dioxygenases, which regulate DNA modification and histone demethylation [9]. Currently, the IDH1 inhibitor ivosidenib is being tested in more than 180 CCC patients with *IDH1* mutation in the phase III trial ClarIDHy [10].

In our study, the majority of the patients (*n* = 28) showed EGFR expression. It has been shown that an activation of EGFR receptors leads to the activation of downstream mitogen-activated protein kinase (MAPK)/ERK and phosphatidylinositol 3-kinase (PI3K)/PTEN/AKT pathways, both of which are important oncogenic pathways in BTC [11]. The data regarding the application of anti-EGFR monoclonal antibodies in advanced, inoperable, or metastatic BTC are contradictory. The three phase II trials BINGO, PICCA, and Vecti-BIL tested the anti-EGFR monoclonal antibodies cetuximab or panitumumab in combination with gemcitabine and a platinum-based agent; however, these agents failed to prolong overall survival [12–14]. In contrast, a phase II trial by Gruenberger et al. [15] published in Lancet and a phase II trial by Rubovszky et al. [16] demonstrated the efficacy of cetuximab in advanced, inoperable, or metastatic BTC.

Similarly, erlotinib, an anti-EGFR tyrosine kinase inhibitor, was tested with and without gemcitabine and oxaliplatin in a randomized, phase III study. Similar to cetuximab or panitumumab, erlotinib did not significantly improve overall survival compared to chemotherapy alone [17].

One reason for the failure of these clinical trials may be that all the patients with different types of BTC are pooled together without taking into consideration the highly variable inter- and intratumoral genetic and epigenetic heterogeneity of BTC. Moreover, the KRAS status of the BTC patients was only evaluated in PICCA, Vecti-BIL, and the phase II trial by Rubovszky. Notably, as shown by Chen et al. the KRAS status is of pivotal importance for the efficacy of EGFR antibodies [18].

Over 80% of the patients expressed p-mTOR, which is an integral part of the PI3K/AKT/mTOR pathway. Its function is pivotal for the regulation of cellular metabolism, growth, angiogenesis, and survival. Deregulation of this pathway plays a key role in the oncogenesis of BTC [19]. In phase II RADiChol, Lau et al. demonstrated a remarkable antitumor activity of the mTOR inhibitor everolimus that was administered as the first-line monotherapy in advanced BTC. The study enrolled 27 patients, and the DCR at 12 weeks was 48%, median progression-free survival (PFS) was 5.5 months, and median OS was 9.5 months [20]. The authors stated that IHC staining for PI3K/AKT/mTOR pathway did not significantly correlate with outcome and the clinical outcomes. However, this study lacked statistical power to determine the potential of mTOR expression to predict everolimus sensitivity. Further, based on the multicenter phase II ITMO study, everolimus demonstrated antitumor activity in 39 BTC patients progressing after prior systemic chemotherapy. The DCR was 44.7%, and the ORR was 5.1%. Everolimus was recommended for two patients. Both of them were PTEN deficient. Several studies showed that mTOR inhibitors have an increased antitumoral activity in cancer patients with loss of PTEN [21]. One of the patients harbored a PIK3CA and a KRAS mutation and had an expression of progesterone receptor. A study by Yi et al. [22] suggests that PIK3CA mutation may be a potential biomarker of sensitivity to everolimus. Thus, everolimus was recommended in combination with an aromatase inhibitor. The other patient was KRAS wild type and displayed an overexpression of EGFR. Consequently, everolimus was offered in combination with cetuximab.

Ponatinib is an oral multitargeted tyrosine kinase inhibitor, that is a pan-FGFR inhibitor and was recommended in one patient with a *FGFR2* mutation and in another with *FGFR* fusion gene. In a pilot study, ponatinib is being tested in 12 BTC patients with *FGFR* fusions (NCT02265341).

One patient with *BRAF V600E* mutation was included in the ROAR trial. Dabrafenib and trametinib are examined in this phase II basket trial in various cancers with *BRAF V600E* mutation, including BTC (NCT02034110). Recently, Wainberg et al. [23] presented a cohort analysis of this trial of 35 BTC patients with *BRAF V600E* mutation. The results were promising: Partial responses and stable disease were reported in 42% and 45% of the patients, respectively.

In a phase II study conducted in South Korea, sunitinib was administered as a second-line treatment in advanced BTC (NCT01082809) and showed marginal efficacy in the Asian population with a time to progression of only 1.7 months [24]. The results of a similar study conducted in Europe are awaited (NCT01718327).

Trastuzumab was recommended in one BTC patient with HER2 expression. In a retrospective analysis, Javle et al. evaluated the application of HER2/neu-directed therapy (trastuzumab, lapatinib, or pertuzumab) in 14 patients with biliary tract cancer, of whom eight persons had a HER2/neu gene amplification or overexpression. One patient experienced complete response, two had a partial response, and three patients achieved stable disease under HER2/neu-directed therapy [25]. Presently, the efficacy of trastuzumab is evaluated in the phase II BILHER trial (NCT03613168).

Trastuzumab emtansine was suggested for one patient with a highly expressed HER2. Preclinical data showed promising results of the antitumor activity of trastuzumab emtansine in HER2-positive BTC cell lines [26].

In the important phase II KEYNOTE-158 trial, the immune checkpoint inhibitor pembrolizumab was administered to 104 advanced BTC patients. Pembrolizumab achieved a partial response in six patients, resulting in an ORR of 5.8%. Median PFS was 2.0 months, and median OS was 9.1 months [27]. Currently, this checkpoint inhibitor is being tested in combination with cisplatin and gemcitabine in the phase II ABC-09 trial (NCT03260712).

The combination therapy is a promising field. Recently, many new multikinase inhibitors and immune checkpoint inhibitors are tested in combination with systemic chemotherapy in metastatic BTC. This treatment strategy may lead to blockage at multiple sites and yield better results [28].

Taken together, the extremely complex tumor biology and spatial and temporal heterogeneity in BTC genetics pose unique challenges for the management of BTC and for drug development. Further research is required to better comprehend the tumor biology.

This study has several limitations. It was a non-randomized, retrospective subanalysis of the single-center MONDTI cohort. In total, a limited number of patients (30 patients) with metastatic BTC were identified. This group lacked an adequate control group, and all patients had good performance status (ECOG 0–1). However, to the best of our knowledge, this is the first study that demonstrates and discusses the potential of PCM in a real-world setting.

This subgroup analysis shows that profiling of BTC is possible and feasible and compatible with the daily clinical routine. PCM in BTC has the potential to become more widely used in cancer drug development and therapy planning and strategy [28–30]. The results and findings of this study need to be confirmed and validated in well-designed clinical trials before they can be recommended its use in clinical practice.

## Electronic supplementary material

Below is the link to the electronic supplementary material.

Supplementary material 1 (DOCX 17 kb)
